# Postoperative X-ray and CT measurement of mounting parameter accuracy for hexapod external fixator in treating tibial fractures: a retrospective study

**DOI:** 10.1186/s12891-026-09593-4

**Published:** 2026-02-07

**Authors:** Zhiming Zhao, Zhao Liu, Yuanyuan Geng, Jian Chen, Chengkuo Cai, Guoqi Ji, Weiguo Xu

**Affiliations:** 1 Department of Traumatic Orthopaedics, Tianjin Hospital, Tianjin, China; 2 Department of Radiology, Tianjin Hospital, Tianjin, China

**Keywords:** Tibial fractures, External fixators, Computed tomography, Radiography, Accuracy

## Abstract

**Background:**

The hexapod external fixator (HEF) allows for precise three-dimensional reduction of tibial fractures, but its therapeutic efficacy is highly dependent on the accuracy of postoperative mounting parameters. Currently, X-ray and computed tomography (CT) are the primary imaging modalities, each with distinct trade-offs between accuracy and efficiency in clinical use. This study compares the accuracy of postoperative X-ray and CT in measuring mounting parameters for HEF in tibial fracture treatment and assesses the associated clinical outcomes.

**Methods:**

This single-center retrospective cohort study included 71 patients with tibial fractures treated with HEFs at our institution between June 2021 and June 2023. The cohort consisted of 40 males and 31 females, aged 30 to 60 years. Patients were divided into two groups based on the imaging method used for postoperative measurement of the hexapod fixator’s mounting parameters: the X-ray group (*n* = 34, using 2D measurements from standard anteroposterior (AP) and lateral radiographs) and the CT group (*n* = 37, using CT scans and 3D reconstruction). Baseline characteristics—including age, sex, mechanism of injury, AO/OTA fracture classification, and Gustilo-Anderson classification—were comparable between groups (all *P* > 0.05). Primary outcomes were the number of electronic prescriptions, time to fracture reduction (from the first postoperative electronic correction prescription to radiographic confirmation of satisfactory reduction), and measurement operation time. Secondary outcomes included final radiological outcome, time to fracture union, and Johner-Wruhs score at final follow-up.

**Results:**

All 71 patients were followed up for a mean of 24.5 months (range: 18–36 months). The number of electronic prescriptions was lower in the CT group (median [IQR]: 1 [1]-[1]) than in the X-ray group (2 [1-2]). Time to fracture reduction was shorter in the CT group (3.3 ± 0.6 days vs. 4.8 ± 0.8 days). Measurement operation time was shorter in the X-ray group (12.9 ± 2.1 min vs. 14.1 ± 1.5 min). All these between-group differences were statistically significant. In the CT group, 81.1% (30/37) achieved satisfactory reduction with a single prescription, significantly higher than the 55.9% (19/34) in the X-ray group (*P* < 0.05). No statistically significant group differences were seen in time to fracture union (X-ray: 26.1 ± 3.2 weeks, CT: 25.7 ± 2.3 weeks), final radiological outcomes (displacement and angulation on AP and lateral views), or Johner-Wruhs scores (excellent and good rate: 82.4% for X-ray, 89.2% for CT; *P* > 0.05). No severe vascular or nerve injuries occurred in either group.

**Clinical trial number:**

Not applicable.

**Conclusion:**

Both X-ray and CT can successfully guide hexapod fixator correction for tibial fractures. CT measurement was associated with greater efficiency in the correction process, requiring fewer adjustments and less time to achieve reduction. However, this did not lead to differences in final radiographic or functional outcomes. The decision to use CT should therefore balance its potential for streamlining the early correction phase against considerations of cost, radiation exposure, and local resources. For many routine cases, X-ray-based measurement remains a robust and effective standard approach.

## Background

Tibial fractures represent a frequent and formidable challenge in clinical orthopaedics. When stemming from high-energy trauma, these injuries are often characterized by severe comminution and displacement, compounded by extensive soft-tissue compromise. This combination of bony and soft-tissue devastation creates a complex therapeutic dilemma, demanding meticulous surgical planning and advanced reconstruction strategies to achieve optimal functional recovery [1, 2]. The core objective of treatment is to achieve precise fracture reduction to restore the normal alignment, length, and rotational alignment of the limb, thereby maximizing functional recovery and preventing long-term complications [3, 4]. As an advanced hexapodal external fixation system, the HEF, which incorporates computer-assisted planning, enables minimally invasive, precise three-dimensional reduction of complex fractures and is increasingly prevalent in clinical practice [5–7].

**Fig. 1:**
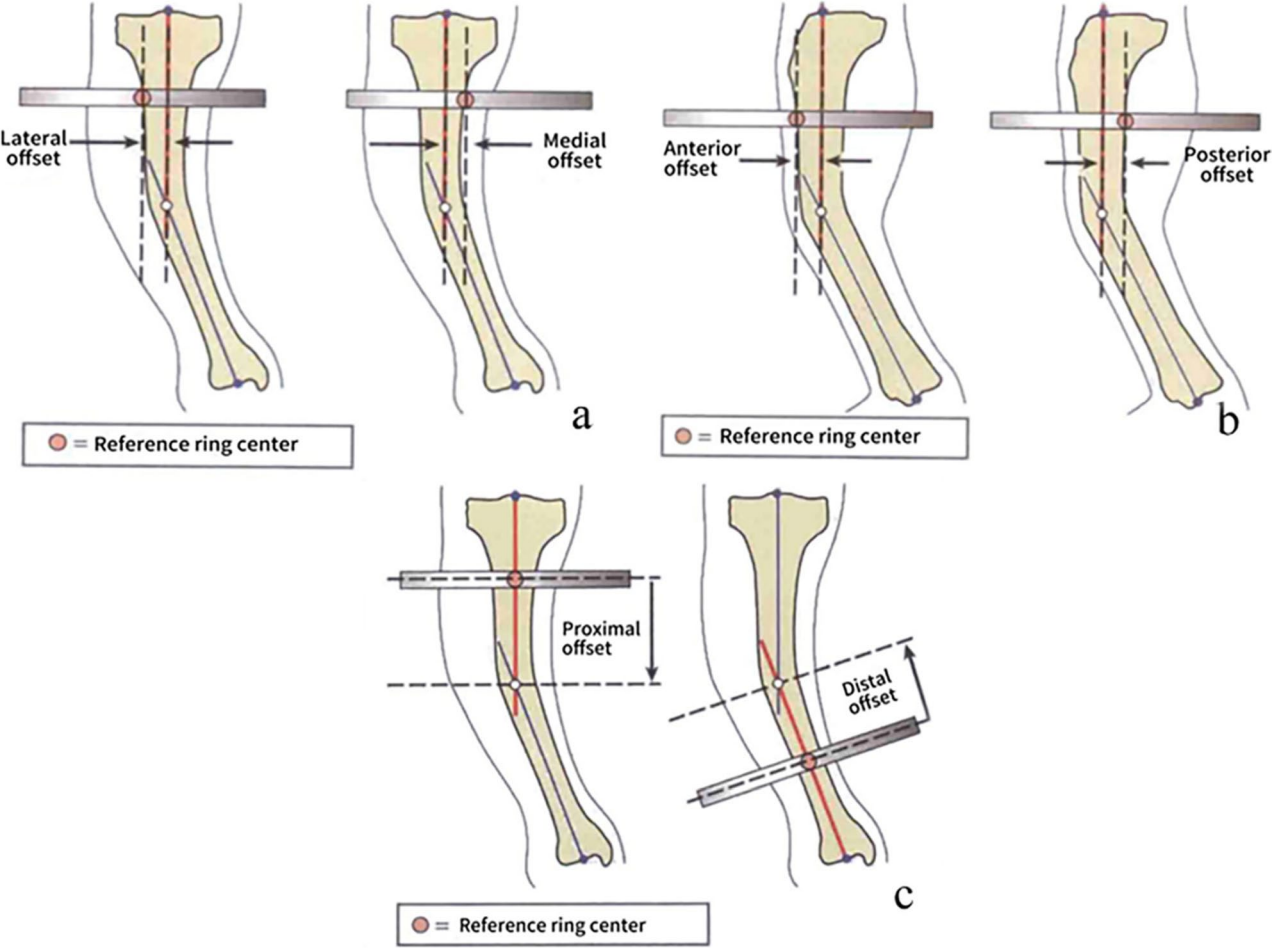
Spatial positional relationship between the reference ring center and the origin. **a** AP offset of the reference ring. **b** Lateral offset of the reference ring. **c** Axial offset of the reference ring

The application of a HEF is guided by a sophisticated software workflow, which generates a corrective electronic prescription based on 13 distinct parameters categorized into three groups: deformity, mounting, and frame parameters. The process of acquiring these parameters is multifaceted. Deformity parameters are derived from a comprehensive analysis of postoperative radiographs and clinical examinations. In contrast, frame parameters, such as the specific model and strut lengths, are predetermined and can be directly entered into the system. Mounting parameters, however, are unique in that they must be meticulously measured from postoperative imaging to ensure accuracy. These parameters define the spatial positional relationship of the reference ring’s center relative to a preset origin (typically the midpoint of the diaphysis at the fracture site), and specifically include mediolateral offset on the AP view, AP offset on the lateral view, proximal-distal offset, and rotational offset on the axial view [8] (Fig. 1). Ideal frame placement should position the reference ring in a neutral position, with an axial rotational offset of 0° [9]. However, the literature reports that approximately one-third of patients still have residual deformity after the execution of the first electronic prescription, with the primary cause being measurement errors in the mounting parameters [10]. To address these residual deformities, remeasurement and input of new parameters are required to generate subsequent prescriptions, which not only increase the patient’s radiation exposure but also prolong the treatment cycle [11]. Therefore, the measurement accuracy of the mounting parameters directly affects the quality of fracture reduction and treatment efficiency [10].

Clinically, the acquisition of mounting parameters predominantly relies on two imaging modalities: X-ray and CT [11, 12]. While conventional X-ray is widely used, CT scanning offers a distinct advantage by generating non-overlapping three-dimensional reconstructions of both the bone and the fixator. This capability facilitates precise measurements across multiple planes and, theoretically, yields mounting parameters of superior accuracy [11]. However, it remains to be determined whether the theoretical precision of CT translates into superior clinical outcomes in terms of treatment workflow efficiency or final functional recovery, given the lack of high-quality comparative studies.

Therefore, this study endeavors to conduct a systematic comparison of postoperative X-ray versus CT measurements for guiding tibial fracture treatment with a HEF, employing a retrospective cohort analysis. We will focus on key performance indicators—including the number of electronic prescriptions required, time to fracture reduction, and the ultimate quality of reduction—to establish an evidence-based framework for informing the optimal clinical measurement strategy.

## Methods

### Patients

This was a single-center, retrospective cohort study. The study protocol was approved by the Ethics Committee of Tianjin Hospital(approval no. 2023 − 168), and informed consent was obtained from all patients. We consecutively included patients with tibial fractures who were treated with a HEF in the Department of Orthopedic Trauma at our hospital from June 2021 to June 2023. The inclusion criteria were: (1) age 30–60 years, (2) AO/OTA type 42 tibial fractures, (3) closed or Gustilo-Anderson type II, IIIA, or IIIB open fractures, and (4) the HEF was used as the definitive fixation method. Exclusion criteria included: (1) pathological fractures, (2) previous fracture or surgery history of the ipsilateral tibia, (3) concomitant knee or ankle fractures affecting limb alignment, and (4) incomplete follow-up data or a follow-up period of less than 18 months. We established this minimum follow-up duration to ensure a comprehensive assessment of long-term functional outcomes. 18-month follow-up provides a sufficient buffer to evaluate stable functional recovery (Johner-Wruhs score) and to monitor for potential delayed complications following frame removal.

The final analysis encompassed 71 patients, with a mean age of 40.8 ± 10.7 years (40 males and 31 females). For the purpose of this study, patients were stratified into two cohorts based on the imaging modality utilized for postoperative measurement of mounting parameters: an X-ray group (*n* = 34) and a CT group (*n* = 37). The assignment to either the X-ray or CT group was not randomized but followed the clinical routine during the study period. The selection of imaging modality was primarily based on a combination of factors: (1) Surgeon’s evolving practice: In the earlier phase of the study, X-ray was the default modality; as familiarity with CT-based measurement increased, CT was more frequently utilized for complex cases. (2) Fracture complexity: CT was generally preferred for fractures with anticipated difficulty in parameter measurement using plain radiographs, such as those with severe comminution, or when the external fixator rings were positioned in close proximity to joints. (3) Practical considerations: These included the availability of CT scanning, the patient’s clinical condition (e.g., ability to undergo CT), and, in some cases, patient concerns regarding radiation exposure or cost. Despite this pragmatic, non-randomized allocation, a post-hoc comparison of baseline characteristics confirmed that the two groups were balanced in terms of age, sex, mechanism of injury, AO/OTA fracture classification, and Gustilo-Anderson classification (all *P* > 0.05), supporting their comparability for the outcomes of interest.(Table 1).

**Table 1:** Comparison of baseline characteristics between the two groups

Variable	X-ray(*n* = 34)	CT(*n* = 37)	*P*-value
Age (year)	42.2 ± 7.3	39.5 ± 8.5	0.154
Gender			0.580
Male	18(52.9)	22(59.5)	
Female	16(47.1)	15(40.5)	
Injury mechanism			0.754
Traffic accident injury	19(55.9)	23(62.2)	
High falling injury	11(32.4)	9(24.3)	
Crushing injury	4(11.7)	5(13.5)	
Injured bone			0.919
Left tibia	16(47.1)	19(51.4)	
Right tibia	18(52.9)	18(48.6)	
AO/OTA grading			0.952
42-A1	7(20.6)	5(13.5)	
42-A2	6(17.6)	7(18.9)	
42-A3	8(23.5)	8(21.6)	
42-B2	5(14.7)	8(21.6)	
42-B3	4(11.8)	4(10.8)	
42-C3	4(11.8)	5(13.5)	
Gustilo-Anderson grading			0.420
Type II	3(8.8)	5(13.5)	
Type IIIA	13(38.2)	9(24.3)	
Type IIIB	7(20.6)	10(27.1)	
Open/closed fracture			0.802
Open	23(67.6)	24(64.9)	
Closed	11(32.4)	13(35.1)	
Soft-tissue condition in closed fractures^༊^			
Tension blisters	6 ( 54.5%)	8 (61.5%)	
Extensive skin abrasions	5 (45.5%)	5(38.5%)	

Data are expressed as the mean ± SD or frequency (%)

༊ Data for closed fractures are presented as n (% of closed fractures in each group)

*The presence of significant tension blisters or extensive abrasions was a key factor precluding immediate internal fixation and favoring definitive hexapod external fixation

### Rationale for hexapod fixator use

The decision to employ a hexapod external fixator as definitive treatment was guided by a holistic assessment prioritizing soft-tissue management and the need for precise correction. Indications included: (1) High-energy open fractures (Gustilo type II, IIIA, IIIB) requiring staged management; (2) Closed fractures with severe soft-tissue compromise, such as significant tension blisters or extensive skin abrasions (see Table 1), where immediate internal fixation was deemed unsafe due to high risk of wound complications; and (3) Fracture patterns benefiting from gradual, multiplanar correction, even in simpler AO/OTA types (e.g., those with substantial initial displacement or metaphyseal involvement). This rationale explains the inclusion of patients with simpler fracture configurations but complex soft-tissue envelopes.

### Preoperative Preparation

For patients with open tibial fractures, emergency debridement and calcaneal traction were performed first, followed by a second-stage HEF fixation once the soft-tissue conditions had stabilized.

### Surgical technique

All procedures were performed by a single surgical team. Each patient was positioned supine on a radiolucent operating table and received either spinal or general anesthesia, with the affected limb appropriately draped and prepared for surgery. A tourniquet was not used intraoperatively.

The procedure commenced with the placement of one to two rings at the proximal and distal aspects of the tibial fracture. These rings were meticulously oriented to achieve maximal perpendicularity to the tibial longitudinal axis. Each ring was then secured using two tensioned, crossing 2.0 mm olive wires, supplemented by one to two 6.0 mm hydroxyapatite-coated half-pins for enhanced stability. Subsequently, a preliminary reduction of the tibial fracture was performed under C-arm fluoroscopy. Manual traction was applied to restore approximate limb length and alignment, while simultaneously correcting angular and rotational deformities. Finally, the six struts were connected and locked to complete the frame assembly.

The intraoperative procedures were identical for patients in both the X-ray and CT groups.

### Measurement methods for mounting parameters

**Fig. 2:**
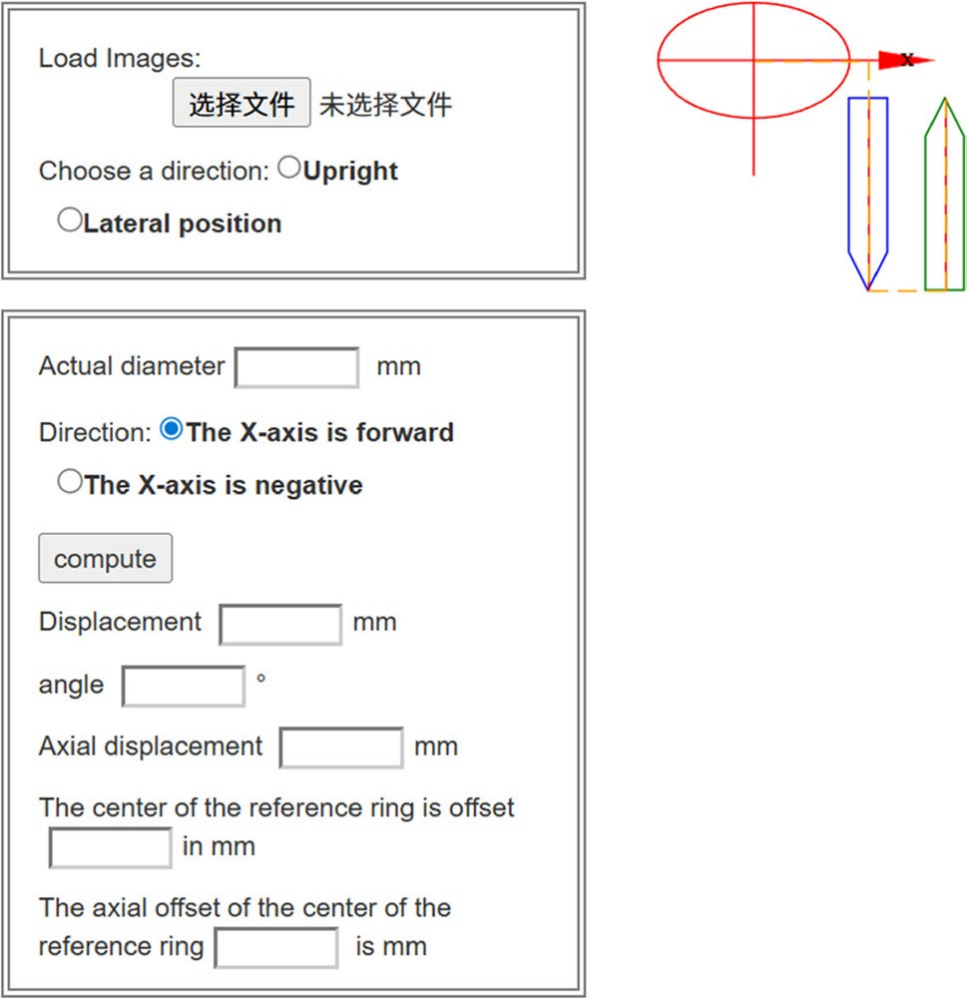
Carefix orthopedic medical correction software - mounting parameters workspace

**Fig. 3:**
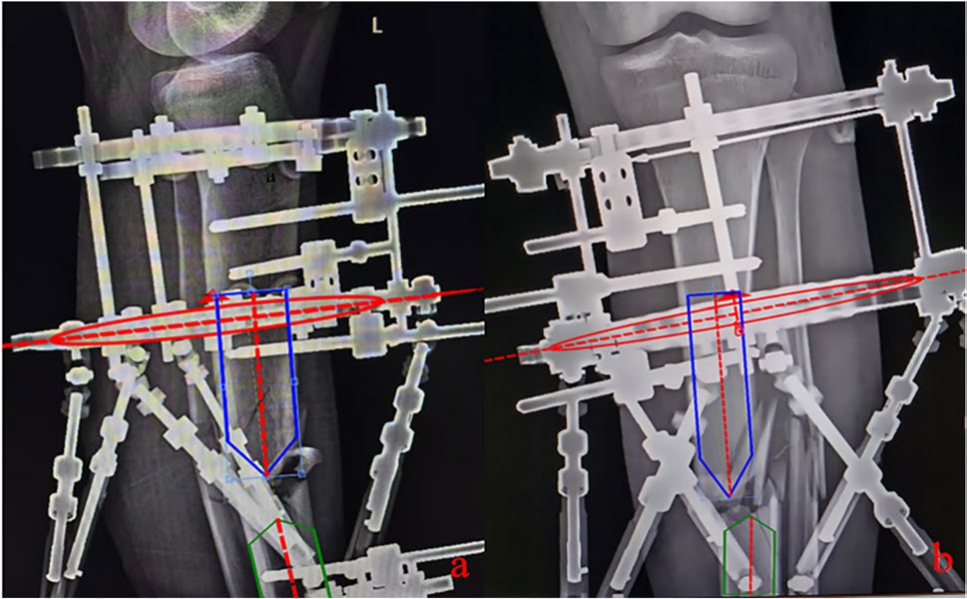
X-ray measurement of hexapod external fixator mounting parameters. **a** The elliptical and arrow markers were aligned with the corresponding rings and anatomical markings on the AP tibial X-ray images. **b** The elliptical and arrow markers were aligned with the corresponding rings and anatomical markings on the lateral tibial X-ray images

In the X-ray measurement group, standard AP and lateral radiographs of the tibia were acquired postoperatively. These images were then individually imported into the mounting parameters workspace of the Carefix orthopedic medical correction software (Fig. 2). Within this workspace, the elliptical and arrow markers were moved to the appropriate positions on the X-ray images (Fig. 3). The system then automatically calculated the mounting parameters based on these rings and anatomical markings.

**Fig. 4:**
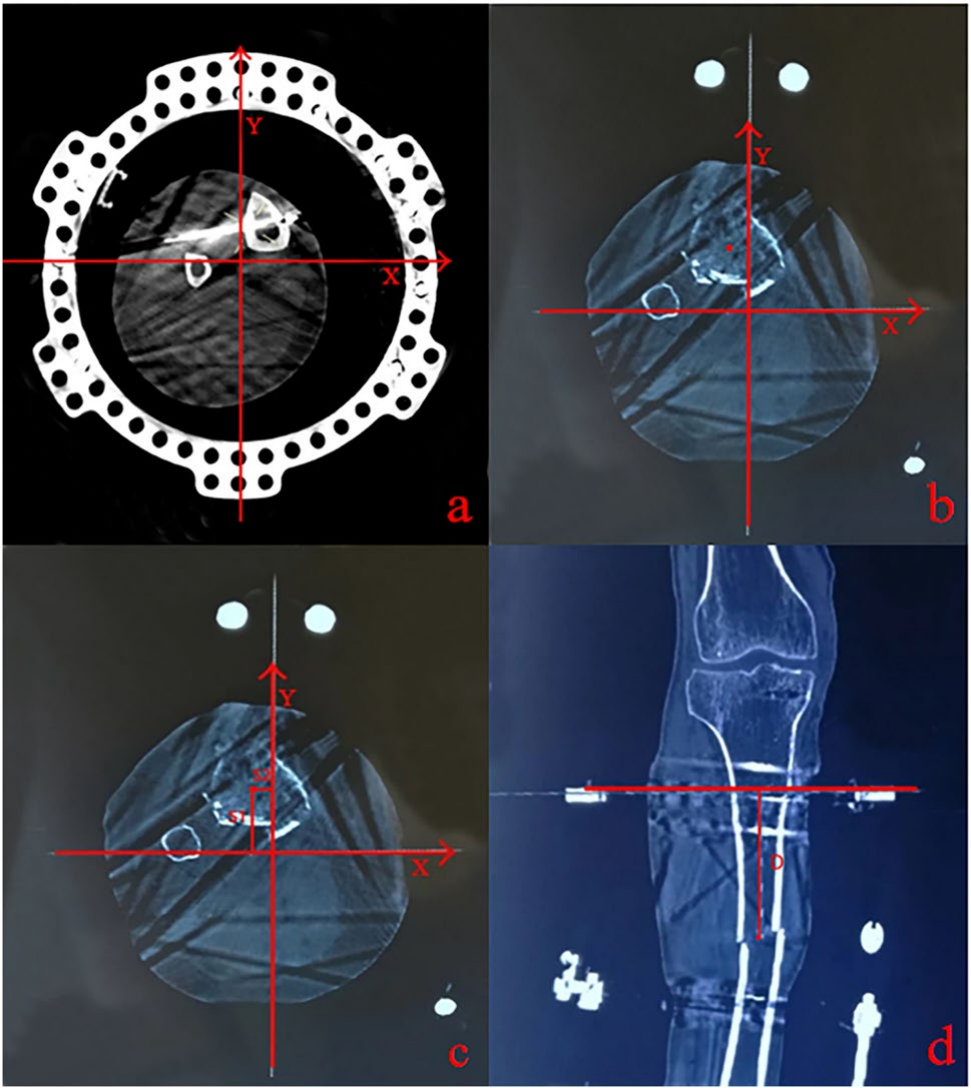
CT measurement of hexapod external fixator mounting parameters. **a** On the slice where the proximal reference ring is fully visualized in the CT workstation, its center was identified. Spatial coordinate axes for the AP and mediolateral directions were then established across all planes. **b** The image was navigated to the level of the fracture line, and the midpoint of the diaphysis (red dot) was located. **c** The distances from the diaphyseal midpoint to the X and Y axes (S1 and S2) were measured, representing the AP and lateral mounting parameters, respectively. **d** On the coronal image, the vertical distance (D) between the fracture line and the reference ring was measured, which represents the axial mounting parameter

For the CT measurement group, a thin-slice CT scan of the affected limb was performed following HEF fixation. Image processing and measurement were conducted on a dedicated CT imaging workstation. Using a 155 mm full ring as an example of a reference ring, its center was identified on the slice where the ring was fully visualized. Spatial coordinate axes for the AP and mediolateral directions were established across all planes. The image was then navigated to the level of the fracture line to locate the midpoint of the diaphysis (origin). The distances from this origin to the coordinate axes (S1 and S2) were measured, representing the AP and lateral translational mounting parameters, respectively. On the coronal reconstruction, the vertical distance (D) between the fracture line (origin level) and the reference ring plane was measured, representing the axial (proximal-distal) mounting parameter (Fig. 4).

In addition to these translational offsets, the angulation of the reference ring relative to the anatomical axis was assessed. The coronal and sagittal angulations of the ring plane were determined by measuring the angle between the ring plane and the anatomical axis on multiplanar reconstructed images. This assessment ensures that the ring is positioned as perpendicular as possible to the mechanical axis, which is an important aspect of frame application [9]. Rotational offset—a deformity parameter reflecting the torsion of the bone segments—was calculated separately based on the alignment of the proximal and distal segments, as described below.

Deformity parameter acquisition: For both groups, the deformity parameters (angulation, translation, rotation, and length discrepancy) required for the correction prescription were derived from a standardized assessment. This assessment combined postoperative full-length AP and lateral radiographs of the tibia with clinical examination (e.g., assessment of rotational profile). In the CT group, while the mounting parameters were obtained from the CT scan as described above, the deformity parameters were still primarily calculated from the plain radiographs to maintain consistency with the X-ray group and with standard clinical workflow. However, the availability of CT images provided an additional reference for verifying complex rotational deformities.

All mounting parameters for both imaging modalities were measured by the same two experienced surgeons (ZL and CKC), each with over 5 years of expertise in hexapod fixator management. Prior to the study, they received standardized training—specifically on the Carefix software for X-ray measurements and the CT workstation protocol—to ensure proficiency and consistency. To maximize reliability and minimize subjectivity, parameters from both X-ray and CT images were measured independently by the two observers. The final value used for generating each electronic prescription was calculated as the average of their two measurements, and inter-observer agreement was assessed for both methods. The measurement operation time was defined as the time elapsed from image loading to parameter acquisition during a single measurement session. This time was recorded for each case by one of the two observers (ZL or CKC) during their independent measurement process.

### Postoperative rehabilitation management and deformity correction protocol

A standardized rehabilitation protocol was initiated for all patients on the first postoperative day. This program consisted of isometric quadriceps contractions and active range-of-motion exercises for both the knee and ankle joints. From the third postoperative day, partial weight-bearing walking with the affected limb was initiated under the protection of double crutches. Pin tract care was performed daily using 75% ethanol.

Postoperatively, the patient’s deformity and the frame’s mounting parameters were input into the proprietary software to formulate an initial correction plan. The clinician then executed this plan by precisely adjusting the six struts to their prescribed lengths. After each prescription (i.e., after the strut adjustments specified in a given prescription were completed), AP and lateral radiographs of the affected limb were taken to evaluate the deformity correction effect. If residual deformity remained after the first adjustment, the measurement data of this residual deformity were re-entered into the computer software to generate a subsequent electronic prescription and continue the adjustments. This process was repeated until satisfactory fracture reduction was confirmed radiographically.

### Efficacy evaluation indicators

A comparative analysis between the two groups was performed, assessing neurovascular injury, the number of electronic prescriptions generated, time to fracture reduction, measurement operation time, final radiological outcomes, and time to fracture healing. At the final follow-up, functional outcomes of the affected limb were evaluated using the Johner-Wruhs score [13], a comprehensive metric that incorporates parameters of fracture healing, neurovascular status, ambulatory ability, range of motion, pain, and residual deformity. Based on the evaluation results, functional recovery was classified into four grades: Excellent (no pain, no limp, normal activity, angular and rotational deformity of 0–5°, shortening of 0–5 mm); Good (mild pain or limp, slight limitation of activity, angular and rotational deformity of 6–10°, shortening of 6–10 mm); Fair (moderate pain, obvious limp, moderate limitation of activity, angular and rotational deformity of 11–20°, shortening of 11–20 mm); and Poor (severe pain, requires crutches for walking, severe limitation of activity, angular and rotational deformity > 20°, shortening of 20 mm, or nonunion/infection).

### Statistical analysis

Statistical analysis was performed using SPSS 22.0 (IBM Corp, USA). Continuous data were assessed for normality using the Shapiro-Wilk test. Normally distributed data are expressed as mean±standard deviation and compared using the independent samples *t*-test. Non-normally distributed continuous data and ordinal data (e.g., number of prescriptions) are presented as median [interquartile range] and compared using the Mann-Whitney U test. Categorical data were presented as frequencies (percentages) and compared between groups using the Chi-square or Fisher’s exact test. A *p*-value < 0.05 was deemed statistically significant, indicating that the observed difference between groups was unlikely to be due to chance alone. However, statistical significance does not necessarily imply clinical importance. We also reported effect sizes (e.g., mean differences, proportions) to aid in clinical interpretation.

## Results

### Baseline characteristics of the patients

All 71 patients successfully underwent surgery and completed follow-up, with a mean duration of 24.5 months (range: 18–36 months). A comparative analysis of the X-ray and CT groups revealed no statistically significant differences in baseline characteristics, including age, sex, mechanism of injury, injury side, fracture type (AO classification), or Gustilo-Anderson classification (*P* > 0.05), confirming the comparability of the two cohorts (Table 1).

### Primary outcome measures

The CT group demonstrated significant advantages over the X-ray group, requiring fewer electronic prescriptions (median [IQR]: 1 [1]– [1] vs. 2 [1]– [2], *P* < 0.05) and achieving fracture reduction in less time (3.3 ± 0.6 days vs. 4.8 ± 0.8 days, *P* < 0.05). Conversely, the measurement operation time was significantly shorter in the X-ray group compared to the CT group (12.9 ± 2.1 min vs. 14.1 ± 1.5 min, *P* < 0.05) (Table 2).

**Table 2:** Comparison of treatment efficiency, radiological outcomes, and fracture healing time between the two groups

Variable	X-ray(*n* = 34)	CT(*n* = 37)	*P*-value
Number of e-prescriptions (times)	2 [1–2]	1 [1–1]	0.002
Fracture reduction time(d)	4.8 ± 0.8	3.3 ± 0.6	< 0.001
Measurement operation time (min)	12.9 ± 2.1	14.1 ± 1.5	0.008
Radiological results			
Translation in AP view (mm)	1.3 ± 0.8	1.6 ± 0.9	0.141
Translation in lateral view (mm)	1.1 ± 0.7	1.3 ± 0.9	0.298
Angulation in AP view (°)	2.9 ± 0.8	2.7 ± 0.7	0.268
Angulation in lateral view (°)	3.2 ± 0.9	2.9 ± 0.6	0.106
Fracture healing time(week)	26.1 ± 3.2	25.7 ± 2.3	0.550

Data are presented as mean ± SD or median [interquartile range] as appropriate

The *P*-value for the number of e-prescriptions is derived from the Mann-Whitney U test, other *P*-values are from independent samples *t*-tests

Analysis of the distribution of electronic prescriptions revealed that a single prescription was sufficient to achieve satisfactory reduction in 81.1% (30/37) of patients in the CT group, compared to 55.9% (19/34) in the X-ray group. Consequently, a smaller proportion of patients in the CT group required two or more prescriptions (18.9% vs. 44.1%). This difference in distribution between the groups was statistically significant (*P* = 0.022). Post-hoc analysis of the individual cells revealed that the CT group had a significantly higher proportion of patients achieving reduction with a single prescription compared to the X-ray group (81.1% vs. 55.9%, *P* = 0.022), which directly accounted for the overall significance. (Table 3).

**Table 3:** Comparison of electronic prescription frequency distribution between the two groups [n(%)]

Variable	Satisfactory reduction achieved with one prescription	Satisfactory reduction required two or more prescriptions
X-ray(*n* = 34)	19 (55.9)	15 (44.1)
CT(*n* = 37)	30 (81.1)	7 (18.9)
*P*-value	0.022	

Data are presented as frequency (percentage)

*P*-values were derived from Fisher’s exact test

#### Subgroup analysis by fracture complexity

Given the clinical interest in whether complex fractures benefit more from CT-based measurement, we performed a post-hoc subgroup analysis focusing on patients with AO/OTA type C fractures (n=9). Among these 9 patients, 4 were in the X-ray group and 5 in the CT group. In the X-ray group, 3 out of 4 type C fractures (75.0%) required two or more electronic prescriptions to achieve satisfactory reduction, whereas in the CT group, only 1 out of 5 type C fractures (20.0%) required more than one prescription. This trend, although not statistically significant in this small subgroup (*P* = 0.206, Fisher’s exact test), aligns with the overall study findings and suggests that the efficiency advantage of CT measurement may be particularly relevant for complex fracture patterns.

### Comparison of secondary outcome measures

With respect to fracture healing and final radiological outcomes, both groups achieved favorable and comparable results. The mean time to fracture union was 26.1 ± 3.2 weeks in the X-ray group and 25.7 ± 2.3 weeks in the CT group, a difference that was not statistically significant (*P* > 0.05). The HEF was removed upon fracture healing. The final radiological results showed no statistically significant differences between the two groups in terms of lateral displacement on the AP view (X-ray group: 1.3 ± 0.8 mm, CT group: 1.6 ± 0.9 mm), lateral displacement on the lateral view (X-ray group: 1.1 ± 0.7 mm, CT group: 1.3 ± 0.9 mm), angulation on the AP view (X-ray group: 2.9 ± 0.8°, CT group: 2.7 ± 0.7°), or angulation on the lateral view (X-ray group: 3.2 ± 0.9°, CT group: 2.9 ± 0.6°) (all *P* > 0.05). This indicates that both measurement methods ultimately achieved a similar anatomical alignment (Table 2).

### Functional outcomes and complications

At the final follow-up, both groups demonstrated good functional recovery of the affected limb. According to the Johner-Wruhs score, there was no statistically significant difference in the distribution of outcomes between the groups (*P* > 0.05). The combined rates of excellent and good results were 82.4% (28/34) for the X-ray group and 89.2% (32/37) for the CT group, respectively (Table 4). To address the potential influence of correction efficiency on final outcome and to assess for qualitative bias in defining ‘satisfactory reduction,’ we performed a subgroup analysis. Among patients who achieved an excellent or good functional score, the proportion requiring two or more electronic prescriptions was higher in the X-ray group (9 of 28, 32.1%) than in the CT group (3 of 33, 9.1%; *P* = 0.026). In terms of safety, no severe complications such as neurovascular injury occurred in either group. The incidence of superficial pin tract infection was similar between the groups (3 cases in the X-ray group vs. 4 cases in the CT group).

**Table 4:** Outcomes of Johner-Wruhs scores in the two groups [n(%)]

Variable	Excellent	Good	Fair	Poor
X-ray(*n* = 34)	26(76.5)	2(5.9)	6(17.6)	0(0)
CT(*n* = 37)	30(81.1)	3(8.1)	4(10.8)	0(0)
*P*-value	0.464			

## Discussion

### Main findings and interpretation

The intraoperative fluoroscopy method for measuring mounting parameters, as described by Rozbruch et al. and Gantsoudes et al. [14, 15], is technically demanding and prone to variability due to reliance on C-arm positioning and marker placement. While postoperative X-ray measurement simplifies the process [12], it remains constrained by the limitations of two-dimensional imaging. In contrast, CT-based three-dimensional measurement, as pioneered by Kucukkaya et al. [11], enables direct spatial quantification of the relationship between the reference ring and the origin, overcoming the inherent inaccuracies of 2D imaging. Our study represents the first systematic comparison of postoperative X-ray versus CT-guided HEF treatment for tibial fractures. Our findings demonstrate that CT measurement, despite requiring more time per session, significantly enhances overall treatment efficiency by reducing the number of electronic prescriptions and shortening the time to fracture reduction, attributable to its superior accuracy in capturing three-dimensional mounting parameters. This aligns with prior studies emphasizing the utility of 3D imaging in complex spatial assessments [16, 17]. Notably, both methods ultimately achieved comparable final radiological alignment and functional outcomes, suggesting that X-ray measurement, through iterative adjustments, can attain similar reduction quality—though potentially at the cost of increased cumulative radiation exposure and prolonged treatment duration.

### The critical role of postoperative imaging quality and initial alignment

For complex fractures (AO/OTA type C), the accuracy of the initial prescription is heavily influenced by postoperative radiograph quality and residual deformity after frame application, factors often more critical than the fracture pattern itself. Optimal orthogonal X-ray views that clearly visualize both reference rings are frequently difficult to obtain. Non-orthogonal projections or incomplete ring visualization introduce significant parallax error into 2D measurements. Additionally, substantial residual deformity increases the sensitivity of software calculations to minor mounting parameter inaccuracies. CT overcomes these limitations by providing a three-dimensional dataset independent of projection angle. This ensures consistent visualization of both rings and bone anatomy, allowing for direct coordinate-based measurement not compromised by positioning. This capability to obtain reliable parameters despite suboptimal initial conditions explains the higher first-prescription success rate observed in the CT group.

### Association between 3D CT measurement and treatment efficiency

The clinical utility of CT-based planning is particularly pronounced in complex scenarios where standard radiographic methods fall short. Our results suggest that this technique is especially valuable for cases presenting with large initial deformities, where overlapping bone structures obscure accurate assessment. Furthermore, the precision of CT offers significant advantages for frames spanning a joint, such as foot frames, and for applications near to a joint. In these situations, defining accurate mounting parameters via X-rays can be difficult due to projectional errors. By providing a reliable three-dimensional reconstruction, CT reduces the reliance on repeat frame correction prescriptions and serves as a more accurate and reliable technique for complex limb reconstruction [18].

### Interpretation of prescription frequency and clinical relevance

While our data confirm that CT guidance reduces the median number of correction prescriptions, the clinical significance of this reduction warrants nuanced interpretation. The absolute difference in medians (1 vs. 2 prescriptions) means that a single CT scan, with its inherently higher radiation dose, effectively replaces the need for one additional X-ray-based adjustment in a substantial proportion of patients. This aligns with the efficiency gain observed in reduced time to reduction. Notably, the proportion of patients in our X-ray group requiring two or more total prescriptions (44.1%) exceeded the often-cited ‘one-third of cases’ in previous reports [10]. This discrepancy may be attributable to our cohort including a spectrum of complex fractures (AO/OTA type C) where precise mounting parameter calculation via 2D X-rays is inherently more challenging, or to differences in measurement protocols. This finding further underscores the particular value of 3D CT measurement in complex scenarios. This is further supported by our finding that among patients with excellent/good functional scores, a significantly higher proportion in the X-ray group had required multiple adjustments (32.1% vs. 9.1%), confirming that the iterative ‘stepwise approximation’ process was frequently invoked to achieve the final satisfactory outcome.

The significant difference in first-prescription success rate (55.9% vs. 81.1%) warrants scrutiny regarding its cause. Importantly, the same experienced operators performed measurements for both modalities, and the CT measurement process itself was slightly longer (14.1 vs. 12.9 min). This suggests that the higher success rate of CT is unlikely to be attributable solely to operator bias or a simple trade-off of significantly more time. Instead, it supports the premise that the inherent three-dimensional, non-projectional data provided by CT is the principal factor enabling more accurate initial parameter calculation, particularly in complex spatial scenarios, thereby reducing the dependency on iterative corrections.

### Clinical interpretation of the reduction time difference

The statistically significant shorter time to fracture reduction in the CT group (mean difference: 1.5 days) warrants careful clinical interpretation. While a reduction of approximately 1–2 days may appear modest within the overall timeline of fracture healing (25–26 weeks), its primary importance lies in optimizing the treatment process rather than directly altering the biology of union. The key implication is that patients in the CT group spent less time in a potentially sub-optimal, unreduced state after surgery. This earlier achievement of satisfactory alignment may contribute to a more predictable and efficient correction pathway, reducing the period of mechanical instability and potentially decreasing patient anxiety associated with prolonged adjustment cycles. However, as correctly noted, given that our rehabilitation protocol initiated partial weight-bearing only from the third postoperative day, this time difference is unlikely to have a measurable impact on the immediate functional milestones or the long-term risk of post-traumatic arthritis in isolation. Therefore, the value of this efficiency gain should be considered alongside the concomitant reduction in the number of required imaging studies and adjustments, collectively contributing to a more streamlined and potentially less burdensome treatment experience for the patient.

### Analysis of cases requiring multiple prescriptions in the CT group

Even within the CT group, 7 patients (18.9%) required a second adjustment. Post-hoc analysis revealed that the most common factor was highly comminuted AO/OTA 42-C3 fractures (four cases), where defining a stable anatomic origin remained subjective despite CT imaging. One case resulted from a technical measurement error on the workstation. For the remaining two cases, no single technical or anatomic cause was clearly isolated, suggesting a possible combination of subtle measurement error and the inherent sensitivity of the correction software to minute input variations. Crucially, for these subsequent corrections, residual deformity parameters were derived from standard follow-up radiographs—not a repeat CT—and combined with the original CT-based mounting parameters to generate the new prescription. This underscores that while CT improves initial accuracy, fracture complexity and measurement precision remain pivotal for first-attempt success.

### Clinical decision-making considerations

Although final radiographic and functional outcomes were equivalent, the efficiency gains with CT measurement offer distinct clinical benefits. Achieving reduction faster and with fewer adjustments streamlines the early treatment phase, which may: (1) reduce patient burden (fewer clinic visits and less adjustment-related discomfort); (2) optimize resource use (potentially lower cumulative imaging and planning time despite higher upfront CT cost); and (3) provide particular value in complex cases (e.g., highly comminuted fractures or frames near joints), where accurate X-ray measurement is most challenging and prolonged trial-and-error adjustments are undesirable. Thus, CT’s advantage lies in offering a more predictable and efficient pathway to the same good result, rather than in altering the final outcome.

The findings of this study indicate that clinical decision-making involves a trade-off between the precision of CT measurement and the convenience and accessibility of X-ray. We acknowledge that, apart from the efficiency gains in the early correction phase, X-ray planning remains highly competitive with CT in terms of final anatomical alignment and functional outcomes. Therefore, our intent is not to suggest replacing X-ray as the standard of care, but rather to identify specific scenarios where the additional precision of CT justifies its cost and complexity. Accordingly, we propose a stratified decision-making framework to guide clinical practice. This framework recommends prioritizing CT measurement in the following situations: first, for complex tibial fractures, such as AO/OTA type 42-C fractures; second, for patients who exhibit significant residual deformity (e.g., angulation > 5° or displacement > 10 mm) after the execution of the first postoperative electronic prescription, necessitating a second adjustment. Conversely, X-ray measurement may be considered for simple diaphyseal fractures (e.g., AO/OTA type 42-A), in primary hospitals without CT equipment, for patients who are sensitive to radiation (such as pregnant women), or for patients who cannot afford the cost of a CT scan due to limited financial resources.

This study did not directly quantify cumulative radiation dose, which is an important consideration in clinical decision-making. As noted in the literature, the effective dose for a standard tibial CT scan (approximately 0.2–0.5 mSv) is higher than that for a single AP and lateral radiographic series (approximately 0.01–0.1 mSv) [19, 20]. Therefore, the potential efficiency gains of CT—fewer adjustments and a shorter reduction time—must be weighed against its higher per-scan radiation exposure. Importantly, 44.1% of patients in the X-ray group required two or more adjustment cycles, each necessitating additional radiographic examinations. A formal, prospective comparison of total effective dose between the two pathways is needed to definitively address this balance. Similarly, while CT entails a higher direct imaging cost, its ability to streamline the correction process may reduce overall treatment duration and associated indirect costs. Future studies should incorporate prospective radiation dosimetry and health economic analyses to provide a more complete framework for decision-making.

### Limitations

This study has several limitations. First, its single-center, retrospective design may introduce selection bias, and the non-randomized grouping could not fully control for all potential confounding factors. Second, the total sample size was limited, and the number of cases in the complex fracture subgroup was insufficient, which may affect the statistical power and generalizability of the conclusions. Third, although measurements from both X-ray and CT were performed by two independent observers and averaged to reduce subjectivity, some degree of inter-observer variability may remain, especially in complex fracture patterns. Fourth, we did not quantitatively compare the cumulative radiation dose or economic costs of the two methods, both of which are key factors in clinical decision-making and warrant future study. Finally, several practical barriers to adopting CT measurement were acknowledged but not fully quantified: the manual measurement on a CT workstation is technically demanding and less integrated into surgical workflow than proprietary X-ray software; while cumulative radiation exposure from repeated X-rays may be comparable to a single CT, total effective doses were not prospectively measured; and a formal cost-effectiveness analysis of routine CT use—considering both direct imaging costs and potential savings from shorter treatment—was beyond the scope of this imaging-accuracy study. These aspects of technical complexity, radiation safety, and cost represent important considerations for implementation that our retrospective design could not definitively address.

## Conclusion

Both X-ray and CT can successfully guide hexapod fixator correction for tibial fractures. CT measurement was associated with greater efficiency in the correction process, requiring fewer adjustments and less time to achieve reduction. However, this did not lead to differences in final radiographic or functional outcomes. The decision to use CT should therefore balance its potential for streamlining the early correction phase against considerations of cost, radiation exposure, and local resources. For many routine cases, X-ray-based measurement remains a robust and effective standard approach.

## Data Availability

The data that support the findings of this study are available from the corresponding author upon reasonable request.

## Abbreviations

HEF: Hexapod external fixator
CT: Computed tomography
AO/OTA: Arbeitsgemeinschaft für Osteosythese/Orthopaedic Trauma Association
AP: Anteroposterior
